# Semiannual Imaging Surveillance Is Associated with Better Survival in Patients with Non-B, Non-C Hepatocellular Carcinoma

**DOI:** 10.1155/2015/687484

**Published:** 2015-09-30

**Authors:** Kuniaki Shindo, Shinya Maekawa, Nobutoshi Komatsu, Akihisa Tatsumi, Mika Miura, Mitsuaki Sato, Yuichiro Suzuki, Shuya Matsuda, Masaru Muraoka, Fumitake Amemiya, Mitsuharu Fukasawa, Tatsuya Yamaguchi, Yasuhiro Nakayama, Tomoyoshi Uetake, Taisuke Inoue, Minoru Sakamoto, Tadashi Sato, Nobuyuki Enomoto

**Affiliations:** First Department of Internal Medicine, Faculty of Medicine, University of Yamanashi, 1110 Shimokato, Chuo, Yamanashi 409-3898, Japan

## Abstract

Since it remains elusive whether and how the imaging surveillance affects the survival in patients with non-B, non-C hepatocellular carcinoma (NBNC-HCC), we conducted this retrospective study which investigated the association between the semiannual surveillance prior to HCC diagnosis and the survival in patients with the initial diagnosis of HCC induced by hepatitis B virus (HBV) and/or hepatitis C virus (HCV) infections (*N* = 141) and non-B, non-C etiology (*N* = 30). It was demonstrated that surveillance was less frequently performed in the NBNC-HCC patients compared to that in HCC patients with HBV and/or HCV infections (B/C-HCC patients), and the survival was unfavorable in NBNC-HCC patients. On the other hand, the survival of NBNC-HCC patients with semiannual surveillance was significantly favorable than those patients without semiannual surveillance, and the survival was similar between B/C-HCCs and NBNC-HCCs with semiannual surveillance. In conclusion, though NBNC-HCC patients compared to B/C-HCC patients had poorer prognosis overall, these NBNC-HCC patients with semiannual surveillance had a better survival almost equivalent to the survival of B/C-HCC patients with semiannual surveillance, demonstrating the clinical utility of the semiannual imaging surveillance program for NBNC-HCCs.

## 1. Introduction

Hepatocellular carcinoma (HCC) is one of the most common cancers in the world, being the fifth in terms of incidence and third in the total number of deaths [1]. In Japan, the death number from HCC exceeds 30,000 per year [2, 3], which has been caused predominantly by chronic liver inflammation induced by hepatitis viruses B (HBV) and C (HCV) [4–6]. Recently, on the other hand, the number of nonviral non-HBV, non-HCV (NBNC) HCCs has been increasing, and 20% of newly diagnosed HCCs are classified as NBNC-HCCs in Japan [7]. It is speculated that the main reason for the increase is attributable to the increase of patients with nonalcoholic fatty liver disease (NAFLD) or nonalcoholic liver disease (NASH) associated with the increase of patients with metabolic disorders [8, 9].

As NBNC-HCCs increase, it has been gradually disclosed that the survival of the NBNC-patients is unfavorable compared to that of HCC patients caused by HBV and/or HCV infections (B/C-HCCs). Compared to B/C-HCCs, NBNC-HCCs are often found in a far advanced stage and their tumor sizes are large while their liver functions are rather conserved [10]. However, it was reported that the survival of NBNC-HCC patients was comparable to that in B/C-HCCs if they were found in the early stages of disease [11, 12]. These recent reports suggest that the poor prognosis of NBNC-HCC patients could be attributable to delay in the HCC diagnosis.

Routine imaging surveillance protocols for early detection of HCC are recommended for patients with chronic hepatitis virus infections [13–19]. Actually, many previous studies reported that routine imaging surveillance (ultrasound, dynamic CT, and MRI with gadolinium ethoxybenzyl diethylenetriamine pentaacetic acid (Gd-EOB-DTPA)) was beneficial for the early HCC diagnosis [20–24] in viral hepatitis patients. Based upon the improved survival of liver cirrhosis patients infected with hepatitis B and C viruses, surveillance with imaging studies with every six months, compared to that with longer intervals or no examination, has been recommended for the early HCC discovery in chronic liver disease patients though the importance of the semiannual surveillance has not been established in NBNC-chronic liver disease patients [14, 19, 25].

In this study, we conducted a retrospective analysis to clarify the effect of semiannual surveillance on the survival of NBNC-HCC patients since effective HCC surveillance protocol has been lacking for NBNC-HCCs so far and it is unknown how the imaging surveillance and its interval would influence the survival of NBNC-HCC patients.

## 2. Patients and Methods

### 2.1. Patients

Among the consecutive patients who were admitted to the Yamanashi University Hospital from January 2008 to March 2011, 171 HCC patients for whom information was available regarding the imaging study intervals up to the initial diagnosis of HCC were included in the analysis. HCCs positive for hepatitis B surface antigen (HBsAg) were classified as B-HCC and those positive for HCV antibodies were classified as C-HCC, while HCCs negative for both HBsAg and HCV antibody were defined as NBNC-HCC. In total, 141 patients were classified as B/C-HCC patients (120 C-HCC and 19 B-HCC, two HCCs with B and C) while 30 patients were classified as NBNC-HCC patients. Diagnosis of liver cirrhosis was made histologically or clinically by the occurrence of liver shrinkage, splenomegaly, or ascites or by the presence of esophageal varices.

### 2.2. Surveillance, HCC Diagnosis, and Treatment

As for the imaging modalities used for HCC surveillance, abdominal ultrasound, abdominal dynamic CT scan, and MRI with Gd-EOB-DTPA were included. The final HCC diagnosis was made when typical features of hypervascular HCC were confirmed by one imaging technique (dynamic CT scan, MRI with Gd-EOB-DTPA, or contrast medium-enhanced ultrasound) and the TNM stage was determined. The treatment method for HCC was basically determined according to the Practice Guideline for HCC established in 2009 [19]. Among the therapeutic modalities, surgical resection and radiofrequency ablations (RFA) were classified as curative therapies while transarterial chemoembolization (TACE), systemic chemotherapy, and other palliation therapies including best supportive care were classified as noncurative therapies. Discharged patients were observed as outpatients at the Yamanashi University Hospital with regular imaging studies. Clinical variables, including tumor stages, therapeutic modalities, and surveillance intervals prior to the initial HCC diagnosis, were investigated for their association with the survival rate. According to the Declaration of Helsinki, this study was carried out after approval was obtained by the ethical committee, Faculty of Medicine, University of Yamanashi.

### 2.3. Statistics

Fisher's exact test was used for categorical data and the Mann-Whitney* U* test or Student's* t*-test was used for numerical data. Survival rates were analyzed by Kaplan-Meier curve analysis with the log-rank test. Multivariate analysis was performed using the Cox proportional hazards model. ROC analysis was used to determine the appropriate cutoff value.

## 3. Results

### 3.1. Comparison of Patient Characteristics Based on the Etiology at the Time of HCC Diagnosis

In Table 1, the clinical characteristics of the patients at the time of initial diagnosis are listed according to the status of hepatitis viral infection (NBNC-HCC versus B/C-HCC). There was no statistical difference between the two groups in terms of the patients' age, sex, percentages of liver cirrhosis, Child-Pugh score, platelet count, ALT level, and tumor markers AFP and DCP. However, there was a tendency for NBNC-HCCs to show more advanced disease determined by TNM classification (*p* = 0.06). As to the imaging surveillance, a proportion of patients without receiving any imaging surveillance was significantly higher in NBNC-HCC than B/C-HCC (*p* < 0.01), and a proportion of patients receiving semiannual surveillance was tended to be low in NBNC-HCC than B/C-HCC (*p* = 0.08).

### 3.2. Factors Affecting the Survival of Patients after the Initial Diagnosis of HCC

When univariate analysis was performed to determine the variables affecting the survival of all the HCC patients, non-B, non-C etiology (*p* < 0.01, HR 6.26), DCP ≥ 40 mAU/mL (*p* = 0.02, HR 3.08), AFP-L 3% ≥10% (*p* = 0.03, HR 2.85), and HCC stage III/IV (*p* < 0.01, HR 3.94) were found to be significantly associated with poor survival (Table 2). No evident association was found in variables including Child-Pugh score, platelets, ALT level, and total AFP level. As for the imaging surveillance prior to HCC diagnosis, receiving semiannual surveillance prior to HCC diagnosis was associated with better survival (*p* < 0.01, HR 6.84). On the other hand, patients without curative therapy showed significantly short survival (*p* < 0.01, HR 8.71). In a multivariate analysis, no semiannual surveillance (*p* = 0.02, HR 4.55), NBNC-HCC (*p* < 0.01, HR 4.32), and noncurative therapy (*p* < 0.01, HR 5.93) were extracted as independent variables associated with poor survival (Table 2).

### 3.3. The Correlation of Semiannual Surveillance and Survival

**Figure 1:**
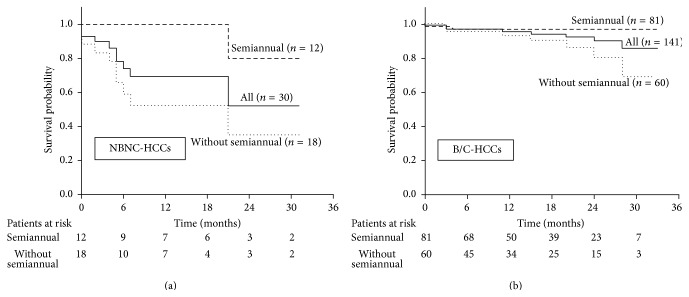
Comparison in the survival of HCC patients with and without semiannual imaging surveillance. Survival Kaplan-Meier curves in all NBNC-HCC patients, in those with semiannual surveillance, and in those without are demonstrated. One-year and 2-year survival in all NBNC-HCC patients are 69% and 52%, respectively, while that in those with semiannual surveillance and that in those without semiannual surveillance are 100% and 80%, and 52% and 35% (*p* < 0.01) (a). Survival Kaplan-Meier curves in all B/C-HCC patients, in those with semiannual surveillance and in those without are demonstrated. One-year and 2-year survival in all B/C-HCC patients are 95% and 90%, respectively, while that in those with semiannual surveillance and that in those without semiannual surveillance are 97% and 97%, and 94% and 80% (*p* < 0.01) (b).

Kaplan-Meier curve analysis was performed to determine whether semiannual surveillance was associated with the survival rate of NBNC-HCC and B/C-HCC patients. As shown in Figures 1(a) and 1(b), the survival was significantly unfavorable in NBNC-HCC patients compared to that in B/C-HCC patients overall (*p* < 0.01, log-rank test). However, the survival was significantly favorable in the NBNC-HCC patients who had received semiannual surveillance than the patients lacking semiannual surveillance (*p* = 0.02, log-rank test) (Figure 1(a)). Likewise, the survival rate was also significantly high in the B/C-HCC patients who had received semiannual surveillance compared to those patients lacking such surveillance (*p* = 0.04, log-rank test) (Figure 1(b)). When the analysis was limited to patients who had received semiannual surveillance, the survival rate was almost comparable between the NBNC-HCC patients and the B/C-HCC patients (*p* = 0.35, data not shown). On the other hand, when the analysis was limited to patients who had not received semiannual surveillance, the survival rate was significantly lower for the NBNC-HCC patients than the B/C-HCC patients (*p* < 0.01, data not shown).

### 3.4. Tumor-Related Factors Associated with Semiannual Surveillance and Disease Etiology

Next, tumor-related factors (DCP, AFP, AFP-L3, tumor size, tumor number, vascular invasion, TNM stage, and therapeutic method) were compared between the NBNC-HCC and the B/C-HCC patients according to the status of the semiannual and nonsemiannual surveillance. As shown in Table 3, when tumor-related factors (DCP, AFP, AFP-L3, tumor size, tumor number, vascular invasion, TNM stage, and therapeutic method) were compared between the NBNC-HCC patients and the B/C-HCC patients among those who had received semiannual surveillance, there was no significant difference between these two groups as to factors except for DCP. However, when tumor-related factors were compared between the NBNC-HCC patients and B/C-HCC patients among those lacking semiannual surveillance, DCP value (*p* < 0.01) and vascular invasion (*p* < 0.01) were significantly worse and more advanced in the NBNC-HCC patients and those factors of tumor size and of HCC stage also tended to be worse in NBNC-HCC patients.

On the other hand, when tumor-related factors were compared between NBNC-HCC patients with and without semiannual surveillance, those tumor-related factors of tumor size, vascular invasion, HCC stage, and curative therapy were significantly unfavorable in those lacking semiannual surveillance.

## 4. Discussion

In this study, we demonstrated that NBNC-HCC patients receiving semiannual imaging surveillance had a significantly better survival than NBNC-HCC patients who lacked such surveillance, and this survival was comparable with that of the B/C-HCC patients with semiannual surveillance.

To date, it has not been clear whether regular, semiannual imaging surveillance would improve the survival of NBNC-HCC patients though establishing the role and protocol of the imaging surveillance in NBNC-HCCs is urgently needed and the importance of imaging surveillance has been suspected from previous studies [7, 10, 11]. Namely, there was no previous study in NBNC-HCC patients demonstrating the importance of imaging surveillance program by analyzing those patients in terms of the correlation between the surveillance and the survival improvement. In this study, compared to B/C-HCC patients, we demonstrated that the proportion of patients without any imaging surveillance was much frequent and the survival was significantly worse in NBNC-HCC patients overall. However, when we compared NBNC-HCC patients with and without semiannual imaging surveillance, we found that the survival was significantly favorable for those with semiannual imaging surveillance than those without. Moreover, the survival in NBNC-HCC patients with semiannual imaging surveillance was almost comparable with that in B/C-HCC patients with semiannual surveillance, disclosing the importance and usefulness of the semiannual imaging surveillance for improving the survival in NBNC-HCC patients (Figures 1(a) and 1(b)). In addition, when the B/C-HCC patients with semiannual surveillance were confined to those without previous antiviral therapy, there was a tendency that the survival of NBNC-HCC patients with semiannual imaging surveillance was rather favorable than that of B/C-HCC patients with semiannual surveillance (data not shown).

In multivariate analysis using the Cox proportional hazard model for the survival in all HCC patients, semiannual imaging surveillance was extracted as an independent determinant with the hazard ratio of 4.55, demonstrating the semiannual imaging surveillance to be significantly related to the improvement of the survival independent of liver disease etiology (Table 2). In B/C-HCCs, the importance of surveillance for the survival improvement has been established from various previous studies, and an interval of 6-month screening has been considered as most appropriate [26, 27]. In this study, though the benefit of surveillance for the survival in NBNC-HCC patients was evident, it is still insufficient to conclude that 6-month interval is the most appropriate interval since patients with surveillance other than 6-month interval were few. However, from log-rank test, difference of the survival was more evident between NBNC-HCC patients with versus without semiannual surveillance than between those patients with versus without any surveillance (data not shown), suggesting the semiannual surveillance would be appropriate while further studies are warranted.

In this multivariate analysis, the etiology of liver disease as NBNC-HCC was also extracted as an independent determinant for the survival. However, we consider that the result does not reflect a biological difference in the malignant potential between NBNC-HCCs and B/C-HCCs, but that the result reflects the poor surveillance status in the NBNC-HCC patients without semiannual surveillance. Namely, the NBNC-HCC patients without semiannual surveillance had a significantly shorter survival than the B/C-HCC patients without semiannual surveillance while no evident survival difference was observed between the NBNC-HCC patients and the B/C-HCC patients if they had received semiannual surveillance (Figure 1). When the surveillance status was investigated further among the patients without semiannual surveillance, 47 out of 60 B/C-HCC patients (72%) had received some surveillance (although the surveillance interval had exceeded 6 months) while only 4 of 18 NBNC-HCC patients (22%) had received the surveillance (data not shown). When tumor-related clinical factors were compared between the NBNC-HCC patients and the B/C-HCC patients, limited to those without semiannual surveillance, NBNC-HCC patients showed more advanced disease in DCP and vascular invasion. Those without semiannual surveillance also tended to have larger tumors and advanced TNM stage (Table 3) while those factors except for DCP were almost comparable between NBNC-HCC patients and B/C-HCC patients if they received semiannual surveillance (Table 3). Collectively, it was considered that cause of the poor prognosis of the NBNC-HCC patients without semiannual surveillance was attributable to insufficient surveillance, resulting in the late HCC diagnosis.

Another important problem in diagnosing NBNC-HCCs is the lack of an appropriate biomarker to diagnose chronic liver disease existing as the background of the NBNC-HCCs [26, 27]. Without the diagnosis of NBNC-chronic liver disease, imaging surveillance cannot be performed. On the other hand, our study demonstrated that 90% (27/30) of NBNC-HCC patients were considered to have liver cirrhosis at the time of HCC discovery judged clinically in most of the patients by the occurrence of liver shrinkage, splenomegaly, or ascites or by the presence of esophageal varices (Table 1). Since an advanced liver fibrosis might underlie HCCs in the liver of NBNC-HCC patients, methods enabling easy and quantitative measurement of liver fibrosis could be candidates to screen and diagnose NBNC-chronic liver disease. In this aspect, several simple methods based on ultrasound or blood biochemical tests such as transient elastography or FIB-4 have been recently developed [28, 29]; these simple and easy modalities might be useful to screen and narrow down the NBNC-HCC candidate patients for the regular imaging surveillance from general population. Actually, only minor proportion of our NBNC-HCC patients was diagnosed as having NBNC-chronic liver disease before HCC development. On the other hand, once the diagnosis of NBNC-chronic liver disease was made, most of these patients received the semiannual imaging surveillance by applying the HCC surveillance guidelines [14, 19, 25] which were made based upon the studies for viral hepatitis.

In conclusion, we demonstrated that semiannual imaging surveillance was significantly associated with the early HCC diagnosis and the favorable survival in NBNC-HCC patients though prospective studies with larger number of patients are still mandatory to confirm our result and to determine the optimal surveillance intervals further.

## Figures and Tables

**Table 1:** Clinical characteristics of the patients.

Variable	NBNC	B/C	*p* value
	*n* = 30	*n* = 141	
Age (yr)^†^	71.0 ± 8.1	68.2 ± 8.9	0.12
Male	73% (22/30)	68% (96/141)	0.57
Accompanied by LC	90% (27/30)	84% (118/141)	0.38
Child-Pugh (A/B, C)	17/10	74/44	0.81
Platelet (×10^4^/mL)^†^	13.0 ± 6.4	11.4 ± 5.2	0.16
ALT (IU/L)^‡^	25 (8–1858)	35 (11–320)	0.45
AFP (ng/mL)^‡^	6.6 (2.2–41719)	14.1 (1.6–150300)	0.76
DCP (mAU/mL)^‡^	48 (9–40753)	22 (7–99760)	0.35
Tumor size (mm)^†^	31.4 ± 19.4	21.8 ± 16.0	<0.01
Tumor number (*n*)^†^	1.8 ± 1.5	1.7 ± 1.3	0.42
Vascular invasion (yes/no)	20% (6/30)	4% (6/141)	<0.01
TNM stage (I, II/III, and IV)	18/12	108/33	0.06
Curative therapy^*∗*^	63% (19/30)	68% (96/141)	0.18
No surveillance	47% (14/30)	12% (17/141)	<0.01
Semiannual surveillance	40% (12/30)	57% (81/141)	0.08

^†^Mean ± SD, ^‡^median (range), and ^*∗*^curative therapy which includes operative resection and radiofrequency ablation.

**Table 2:** Univariate and multivariate analysis for variables associated with survival.

Variable	*n*	Univariate analysis			Multivariate analysis		
		HR	95% CI	*p* value	HR	95% CI	*p* value
Age (years)							
≥71	97	1.66	0.67–4.09	0.27			
≤70	74						
Gender							
M	118	3.43	0.79–14.86	0.08			
F	53						
Etiology							
NBNC	30	6.26	2.54–15.45	<0.01	4.32	1.46–12.8	<0.01
B/C	141						
Child-Pugh							
B, C	30	2.02	0.82–4.98	0.13			
A	141						
Platelet (/mL)							
≤12.0 × 10^4^	104	0.81	0.32–2.02	0.65			
≥12.1 × 10^4^	67						
ALT (IU/L)							
≥40	65	1.08	0.43–2.70	0.87			
≤39	106						
AFP (ng/mL)							
≥20.1	74	1.66	0.67–4.10	0.27			
≤20.0	97						
DCP (mAU/mL)							
≥40	60	3.08	1.23–7.69	0.02	1.14	0.39–3.32	0.82
≤39	106						
AFP-L3 (%)							
≥10.0	34	2.85	1.12–7.27	0.03	1.80	0.62–5.22	0.28
≤9.9	137						
HCC stage (TNM)							
III, IV	45	3.94	1.58–9.81	<0.01	1.48	0.50–4.31	0.48
I, II	136						
Semiannual surveillance							
No	73	6.84	1.98–23.64	<0.01	4.55	1.21–17.08	0.02
Yes	98						
Curative therapy^*∗*^							
No	56	8.71	2.88–26.33	<0.01	5.93	1.88–18.67	<0.01
Yes	115						

^*∗*^Curative therapy includes operative resection and radiofrequency ablation.

**Table 3:** Comparison of tumor-related factors between NBNC-HCCs and B/C-HCCs in each surveillance status.

Variable	Semiannual surveillance		*p* value (I) versus (II)	Without semiannual surveillance		*p* value (III) versus (IV)	*p* value^*∗∗*^ (I) versus (III)
	(I) NBNC	(II) B/C		(III) NBNC	(IV) B/C		
	*n* = 12	*n* = 81		*n* = 18	*n* = 60		
DCP (mAU/mL)							
≥40	6	18	0.04	13	25	0.02	0.22
≤39	6	63		5	35		
AFP (ng/mL)							
≥20.1	3	33	0.30	8	30	0.68	0.28
≤20.0	9	48		10	30		
AFP-L3 (%)							
≥10.0	1	14	0.43	5	14	0.70	0.19
≤9.9	11	67		13	46		
Tumor size (mm)							
≥21	3	16	0.67	16	39	0.051	<0.01
≤20.0	9	65		2	21		
Tumor number (*n*)							
1	8	53	0.93	8	32	0.51	0.23
≥2	4	28		10	28		
Vascular invasion							
Yes	0	2	0.58	6	4	<0.01	0.02
No	12	79		12	56		
HCC stage (TNM)							
III, IV	1	12	0.55	11	22	0.07	<0.01
I, II	11	69		7	38		
Curative therapy^*∗*^							
No	2	27	0.25	9	35	0.53	0.04
Yes	10	54		9	25		

^*∗*^Curative therapy includes operative resection and radiofrequency ablation.

^*∗∗*^Comparison of NBNC-HCCs with versus without semiannual surveillance.
